# Function of exosomes in neurological disorders and brain tumors

**DOI:** 10.20517/evcna.2021.04

**Published:** 2021-03-30

**Authors:** Lan Xiao, Sangeetha Hareendran, Y. Peng Loh

**Affiliations:** Section on Cellular Neurobiology, Eunice Kennedy Shriver, National Institute of Child Health and Human Development, National Institutes of Health, Bethesda, MD 20892, USA

**Keywords:** Extracellular vesicles, neurodegenerative disorders, glioblastoma, Alzheimer’s disease, neurodevelopment

## Abstract

Exosomes are a subtype of extracellular vesicles released from different cell types including those in the nervous system, and are enriched in a variety of bioactive molecules such as RNAs, proteins and lipids. Numerous studies have indicated that exosomes play a critical role in many physiological and pathological activities by facilitating intercellular communication and modulating cells’ responses to external environments. Particularly in the central nervous system, exosomes have been implicated to play a role in many neurological disorders such as abnormal neuronal development, neurodegenerative diseases, epilepsy, mental disorders, stroke, brain injury and brain cancer. Since exosomes recapitulate the characteristics of the parental cells and have the capacity to cross the blood-brain barrier, their cargo can serve as potential biomarkers for early diagnosis and clinical assessment of disease treatment. In this review, we describe the latest findings and current knowledge of the roles exosomes play in various neurological disorders and brain cancer, as well as their application as promising biomarkers. The potential use of exosomes to deliver therapeutic molecules to treat diseases of the central nervous system is also discussed.

## INTRODUCTION

Extracellular vesicles (EV) were first described in 1967 as “platelet dusts” in plasma^[1]^. Currently, EVs are divided into three main categories based on the origination and size: exosome derived from endosomes, ranging from 40 to 100nm in diameter; microvesicle/shedding particles from plasma membrane, which are larger than 100nm in diameter; apoptotic bodies from plasma membrane, which are 1-5 μm in diameter^[2,3]^. In this review, while most of the literature cited has used the term exosome, the degree of characterization of the exosomes in the various papers varied; most have met the guidelines such as size by NTA, exosomal markers and electron microscopy or zeta view, reported for exosomes in MISEV2014^[4]^, and others with insufficient characterization to confirm specific identity as exosomes are referred to as extracellular vesicles.

Exosomes, initially described as vesicles released from various types of cultured cells^[5]^, are microvesicles derived from endosomal membranes. Microvesicles were first described by Dr. C. Turbide in 1987 in his study of maturation of sheep reticulocyte. Vesicles obtained after 100,000x g centrifugation were found to contain some characteristic activity of the reticulocyte^[6]^. These vesicles were then further defined as being originated from endosomes, with a diameter from 30 to 100nm^[7]^. As a subtype of extracellular vesicles with a bilayer membrane that bud from cell membrane and/or are secreted, exosomes are heterogeneous and influenced by the physiological and pathological conditions of the originating cells. Exosomes are distributed broadly in human secretions and act as intercellular messengers via transferring or exchanging DNA, RNA, and proteins between cells^[8,9]^.

Recently, emerging studies have revealed that exosomes have more complicated facets. They are not just secreted as cellular wastes or by-products, but contain a variety of cargos such as proteins, lipids, and nucleic acids, and exert their function by delivering cargoes to target cells and modulating the bioactivity of recipient cells. Therefore, exosomes serve as a new mode of intercellular communication and play a critical role in biological systems, and pathogenesis of diseases, including those of the central nervous system. In addition, the ability of exosomes to cross the blood-brain barrier makes them ideal therapeutic delivery vehicles and potential biomarkers for neurological disorders^[10]^.

### Exosome structure and content

**Figure 1.**
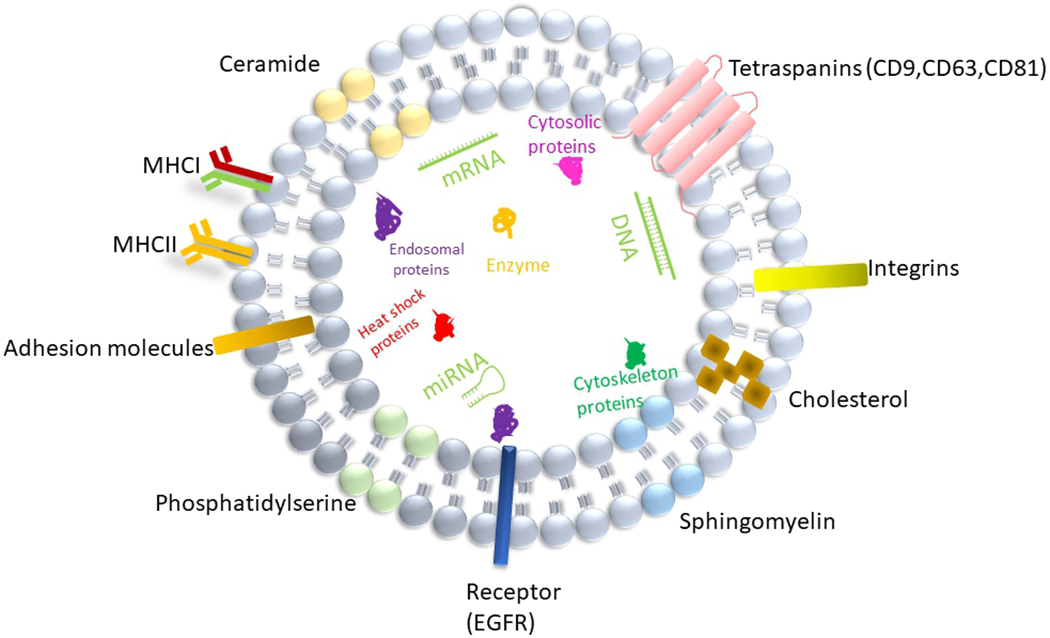
Structure and composition of exosome. Exosome is a lipid bilayer structure that contains lipids, proteins and nucleic acids. Sphingomyelin, phosphatidylserine, cholesterol and ceramides are highly distributed on the membrane. In addition, exosomes also contain a variety of proteins such as major histocompatibility complex I and II (MHCI and MHCII), tetraspanins (CD9,CD63,CD81), endosomal origin proteins (ALIX,Tsg101), heat shock proteins (HSP70,HSP90), enzymes(GAPDH, nitric oxide synthase, catalase), receptor(EGFR), adhesion proteins, integrins, cytoskeleton proteins (actin, gelsolin, myosin, tubulin) and cytosolic proteins, as well as RNA, miRNA and DNA.

Exosomes are released from a variety of cell types, and can be found in physiological fluids such as blood^[11]^, cerebrospinal fluid^[12]^, saliva^[13]^,urine^[14]^ and breast milk^[15]^. Exosomes consist of a wide range of molecules such as proteins, lipids and nucleic acids [Figure 1], and reflect the pathophysiology and physiological features of parental cells.

Current studies have shown that exosome membranes are enriched in sphingomyelin, phosphatidylserine, cholesterol, and ceramides. Exosomes contain a variety of proteins such as tetraspanins (CD9, CD63, CD81), endosomal origin proteins (ALIX, TSG 101), heat-shock proteins (HSP70, HSP90), enzymes(GAPDH, nitric oxide synthase, catalase), receptor (EGFR), major histocompatibility complex I-II, adhesion proteins, integrins, cytoskeleton proteins (actin, gelsolin, myosin, tubulin) and cytosolic proteins^[16,17]^. Irrespective of the origin, certain proteins such as TSG101, HSP70, CD81 and C63 are exclusively involved in the biogenesis of exosomes, and thus generally used as exosome markers. However, since the purity of the exosomes isolated has not been fully assessed in some studies, it is possible that skeletal proteins e.g., actin, myosin and tubulin reported to be present in exosomes may be contaminants of the exosome-enriched fraction. In addition, lipid components within exosomes can be incorporated into recipient cells and mediate complex biological effects^[2]^. Moreover, RNA sequencing showed that mRNA and microRNA are also abundant in exosomes from human plasma, in addition to other species of RNA such as ribosomal RNA, small nuclear RNA, transfer RNA^[2,18]^ and long RNA^[19]^ that maintain critical biological functions.

### Exosome biogenesis, secretion, and uptake

**Figure 2.**
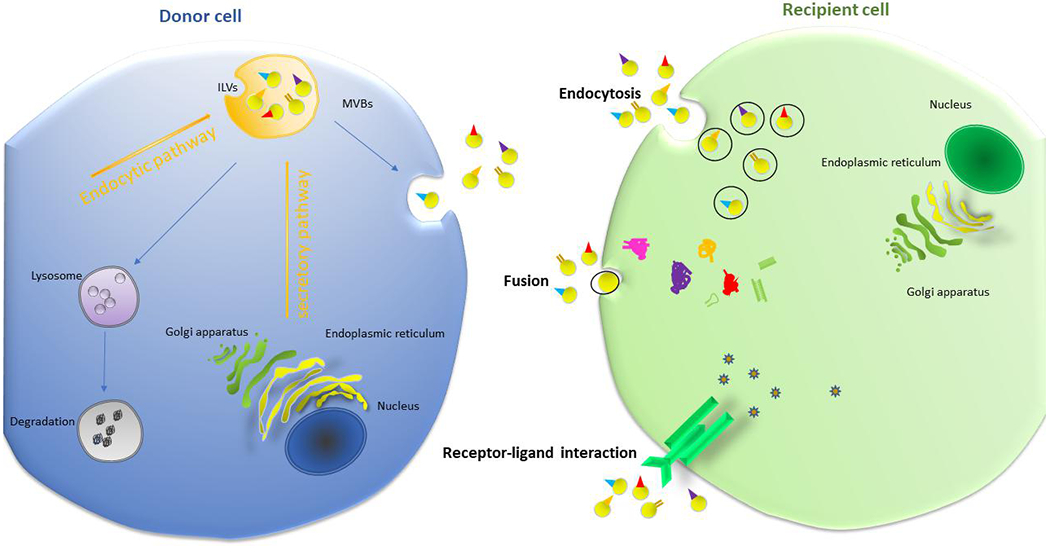
Biogenesis of exosome. The biogenesis of exosomes occurs when multivesicular bodies uptake intraluminal vesicles formed from either endocytic pathway or ER/Golgi secretory pathway. Then, MVBs either fuse with cellular membrane to release exosomes, or fuse with lysosomes for cargo degradation. After releasing into extracellular space, exosomes act as a mediator of intercellular communication through being taken up by recipient cells via endocytosis, fusion or receptor-ligand interaction. This process can be either paracrine or endocrine in manner. MVBs: Multivesicular bodies.

Exosome biogenesis is a complicated process that involves a variety of signaling cascades. Exosomes are formed by multi-vesicular bodies (MVBs) which are late endosomes. The membrane of MVB buds inward to form intraluminal vesicles (ILVs) with components derived from either endocytic pathway or secretory (ER/Golgi) pathway, into the endosomal lumen^[20]^ [Figure 2]. Following accumulation of vesicles, MVBs will be either transported to lysosomes for degradation^[21]^ or fused with plasma membrane to release ILVs into extracellular space as exosomes^[22]^. However, how the cargo is sorted to ILVs and how formation and release of exosomes are regulated by internal and external factors are still not fully understood.

Recent studies indicate that both endosomal sorting complex transport (ESCRT)-dependent and ESCRT-independent pathways are involved in the formation and secretion of exosomes^[2,23]^. ESCRT consists of four major protein complexes, including ESCRT0, ESCRT-I, ESCRT-II, ESCRTIII and associated AAA ATPase Vps4 Complex. In an analysis with RNA interference screen targeting 23 components of ESCRT and associated proteins, it was found that seven ESCRT proteins contributed to the release of exosomes^[24]^: Knockdown of ESCRT-0 proteins Hrs and TSG101, ESCRT-I protein STAM1 decreased the secretion of exosomes; in contrast, knockdown of ESCRT-III proteins CHMP4C, VPS4B, VTA1 and ALIX increased the secretion of exosomes. Further studies revealed that ESCRT-0 sequestered ubiquitinated proteins into specific domains of endosomal membrane, and then combined with ESCRT-III after crosslinking with ESCRT-I and ESCRT-II complex. ESCRT-III finally promotes intraluminal vesicle formation via facilitating the budding process and separation from the MVB membrane^[25]^. Interestingly, syndecan heparan sulphate proteoglycans and their cytoplasmic adaptor, syntenin, have been shown to regulate exosome formation via modulating ALIX through LYPX(n)L motifs to facilitate the intraluminal budding of endosomal membranes^[26]^. These results suggest ESCRT is critical for cargo sorting, multivesicular body formation, and the budding process^[27]^.

Conversely, a large amount of evidence indicates that exosomes can also be formed and released in an ESCRT-independent manner. Studies showed that when four major ESCRT complexes were simultaneously silenced, ILVs were still observed in MVBs, suggesting existence of a ESCRT-independent mechanism^[28]^. In addition, other proteins and lipids are also involved in the regulation of exosome biogenesis and secretion. Tetraspanins, transmembrane proteins that are highly distributed in exosomes, contributed to the ESCRT-independent exosome release^[29]^: overexpression of tetraspanins CD9 and CD82 increased catenin in exosomes released from HEK293 cells^[30]^; tetraspanin Tspan8 promoted recruitment of specific proteins and mRNA into exosomes, such as CD106 and CD49d that are critical for exosome-endothelial cell binding and internalization^[31]^; Tetraspanin CD63 has also been reported to be involved in exosome biogenesis as evidenced by decreased small vesicle secretion after (CRISPR)/Cas9 knockout of the CD63 gene in HEK293 cells^[32]^; finally, tetraspanin-enriched microdomains and tetraspanin CD81 are important for sorting specific receptors and components toward exosomes^[33]^. Furthermore, ceramides have been shown to enhance domain-induced budding due to its activity to promote negative curvature of endosomal membrane^[34]^. Rab guanosine triphosphatases (GTPases) such as Rab27a/b^[35]^, and Rab35 and GTPase-activating proteins TBC1D10A-C have also been reported to contribute to the process of exosome secretion pathway^[36]^.

Interestingly, cellular homeostasis also can affect exosome secretion. For example, increased intracellular Ca^3+^ induced more exosome secretion in K562 cells, a hematopoietic cell line^[37]^. Environmental pH has also been shown to influence exosome secretion^[38]^. In addition, cellular stress such as irradiation^[39,40]^, cisplatin treatment^[41]^, exposure to hypoxia^[42]^ and ER stress^[43]^ can all increase exosome release. Increased release of waste via exosomes might be a natural response to stress, but also could be an approach for cells to communicate with each other under pathological conditions. Particularly, many neurodegenerative disorders are associated with lysosomal or autophagy dysfunctions and aggregations of pathological proteins; exosomes could play a critical role in such neuropathogenesis^[23]^.

As a critical mediator for intercellular communication, exosomes are taken up by recipient cells via three major methods: receptor-ligand interaction, fusion with plasma membrane, and endocytosis by phagocytosis^[17]^ [Figure 2]. For receptor-ligand uptake, the molecular mechanism remains elusive. Current studies revealed that Tim1- or Tim4-expressing Ba/F3 B cells could bind exosomes via phosphatidylserine, suggesting Tim4 and Tim1 are possible phosphatidylserine receptors for exosomes^[44]^. Another study implied that intercellular adhesion molecule 1 (ICAM-1) is critical for mature exosomes to prime naive T cells^[45]^. Fusion with plasma membrane was supported by studies showing exosomes can be taken up by melanoma cells through membrane fusion^[17]^. Interestingly, K562 or MT4 cells-derived exosomes were internalized more efficiently by phagocytes than non-phagocytic cells, implying that phagocytosis may play a unique role in exosome-cell interactions and uptake^[46]^.

Strikingly, numerous studies have indicated that exosomes are critical for communication between different neural cell types. Microglia could specifically internalize oligodendrocyte-derived exosomes by macropinocytosis, and most of these microglia were MHC class II negative and did not activate immunological responses^[47]^. Neurons have been shown to be able to internalize oligodendrocyte-derived exosomes by endocytosis^[48]^. In addition, crosstalk between neuron and glia also occurs through exosomes. Exosomes from stressed astrocytes that were exposed to oxygen and glucose deprivation could produce neuroprotective effects against oxidative stress in neurons and this effect was dependent on Prion protein^[49]^. It has been demonstrated that exosomes are internalized via several mechanisms and the uptake depends on the type of recipient cells. For example, exosomes derived from neuroblastoma bound to neurons and glial cells, but were preferentially endocytosed by glial cells; exosomes derived from cortical neurons were exclusively bound and endocytosed by neurons^[50]^. Indeed, a lot more studies are needed to understand the specificity and molecular mechanism of exosome uptake among different neuronal and glial cell types.

### Exosome-mediated intercellular communication in the nervous system

In 1980, exosomes were still believed to be a means of disposing cell debris^[51]^. However, emerging studies have indicated that exosomes also play multiple roles in biological activities such as cell-to-cell communication, which was traditionally considered to be mediated by gap junction, receptor/ligand, or electrical and chemical signals^[52,53]^. Studies showed that exosome release was increased from cortical neurons by treatment with GABA receptor antagonist, bicucullin; however, this increase was blocked by either AMPA receptor antagonist, CNQX, or NMDA receptor antagonist, MK-801, suggesting exosome release was regulated by glutamatergic synaptic activity^[54]^.

Oligodendrocytes secrete exosomes into extracellular space that can inhibit morphological differentiation in oligodendrocytes and myelin formation, and this effect could be blocked with inhibitors of actomyosin contractility. Interestingly, conditioned neuronal medium dramatically reduced secretion of exosomes from oligodendrocytes, suggesting interaction between neurons and oligodendrocytes during myelin biogenesis^[55]^. Other studies have shown that microglia could internalize exosomes released from oligodendroglia by macropinocytosis, which was then transferred to late endosomes and lysosomes^[47]^. Conversely, studies have revealed that neurotransmitters could stimulate the release of exosomes from oligodendroglial, which subsequently could be internalized and utilized by neurons^[48]^. Mice with absence of proteolipid protein and 2′,3′-cyclic nucleotide 3′-phosphodiesterase, which are enriched in oligodendroglial exosomes, exhibited axonal degeneration^[56]^. In addition, it was shown that Hsp/Hsc70 exiting from oligodendroglia could be taken up by squid giant axon^[57]^, and this process is likely mediated by exosomes^[58]^.

Neurons also regulate intercellular communication and maintain homeostasis such as neurogenesis and synaptic activity via exosomes. Studies using electron microscopy showed that exosomes were secreted from somato-dendritic compartments of mature cortical neurons, confirming neurons secrete exosomes^[54]^. Exosomes released from primary cortical neurons contained several functional proteins that could regulate synaptic activity, and the release of exosomes was controlled by depolarization^[59]^. Cystatin C was detected in exosomes released from mouse primary neurons and played a critical role in neuroprotection^[60]^. In addition, studies in *Drosophila* neuromuscular junction demonstrated that release of exosomal synaptotagmin 4 from presynaptic terminals was crucial for synaptic growth^[61]^. Co-incubation of mouse microglial cell line with PC12 cells enhanced the elimination of degenerating neurites in PC12, and treatment with PC12-derived exosomes significantly increased the pruning activity of microglia^[62]^. In addition, exosomes secreted from primary cortical neurons were internalized into astrocytes and upregulated GLT1 proteins^[63]^.

Microglia can also have crosstalk with neurons and modulate neuronal activity through exosomes. Synapsin I has been observed in the exosomes released from glial cells and found to promote neurite outgrowth in hippocampal neurons and survival of cortical neurons^[64]^. Also a group of miRNA, including miR-146a-5p, has been detected in the extracellular vesicles released from microglia, which regulates the expression of important synaptic proteins^[65]^.

All the evidence suggests that exosomes contribute to intercellular communication via internalization by target cells, activating downstream signaling cascades, or releasing components into the extracellular space. However, the precise understanding of the molecular mechanism underlying this process continues to evolve. Since most experiments were performed *in vitro*, further studies in animal models will open up new perspectives for understanding the function of exosomes in communication in the central nervous system^[48]^.

### Role of exosomes in neurodevelopment

Recent studies have shown that exosomes play an integral role in normal neurodevelopment such as neural plasticity, and contribute to the pathological changes in neurodevelopmental diseases^[66]^. For instance, embryonic cerebrospinal fluid-derived exosomes improved neural stem cell amplification through targeting the rapamycin complex 1 pathway^[67]^. Exosomes also seem to act as a regulator in the niche of mesenchymal stem cell and a modifier of proliferation and differentiation of neural stem cells^[68]^. Exosomes originated from neural progenitor cells have been shown to promote neuronal differentiation and facilitate neurogenesis through miR-21a^[69]^. Exosomes from human induced pluripotent stem cell (hiPSC)-derived neurons increased proliferation in human primary neural cultures *in vitro.* In parallel with *in vitro* studies, injection of exosomes purified from DIV9 rodent primary neural cultures into the lateral ventricles of P4 mouse brains increased neurogenesis in the dentate gyrus of hippocampus^[70]^. On the other hand, studies have shown that exosomes are not only involved in neurogenesis, but also regulate synaptogenesis and neural circuit development. For example, treatment with normal control exosomes could reduce damages in neuronal proliferation, differentiation, synaptogenesis, and synchronized firing in methyl-CpG-binding protein 2 (MECP2)-knockdown human primary neural cultures, which is a key gene contributing to abnormal neurodevelopment in Rett syndrome. Further proteomic analysis revealed that normal (control) exosomes may contain critical factors that are crucial for neuronal maturation and synaptogenesis which are absent in MECP2LF exosomes, suggesting the involvement of exosomes in neuronal development. Interestingly, exosomes have been reported to produce therapeutic effects in neurodevelopment disorders *in vivo.* Intranasal treatment with exosomes derived from mesenchymal stem cells enhanced behavioral autistic-like phenotype such as social vocalization and reduced repetitive behaviors in Shank3B Knockout autism mouse model^[71]^. Recently, extracellular vesicles have been used to encapsulate CRISPR/Cas9 genome editing machinery for delivery to cells. This could be a potentially new approach for delivering Cas9/sgRNA for treating a variety of genetic diseases, including those impacting the nervous system^[72,73]^.

### Function of exosomes in neurodegenerative disorders

As a critical mediator for cell communication, exosomes have been reported to augment the progression of neurodegenerative disorders such as Alzheimer’s disease, Parkinson’s disease, Prion disease, Amyotrophic lateral sclerosis and Huntington’s disease, via delivery of proteins or molecules associated with the pathology of such diseases [Table 1].

### Role of exosomes in Alzheimer’s disease

Alzheimer’s disease (AD) is one of the most devastating neurodegenerative disorders that cause dementia and decreased cognitive function. It currently affects more than 5 million people in the United States and is expected to rise to about 13.8 million by 2050^[74,75]^. Accumulation of amyloid β-peptide (Aβ) plaque extracellularly and formation of neurofibrillary tangles from hyperphosphorylated tau intracellularly are pathological hallmarks of AD that generally precede the clinical symptoms^[76]^. Recent studies have revealed that exosomes play very complex roles in AD^[76–79]^. Both Aβ peptide and tau are released from exosomes and have been implicated in the propagation of aggregates of these proteins. A recent proteomic and bioinformatics study of exosomal proteins in human iPSC neurons expressing mutant Tau (mTau) revealed many differences with normal exosomes such as the presence of a PP2A phosphatase inhibitor. Their data suggest that mTau exosomes may be able to regulate propagation of phosphorylated tau *in vivo* and contribute to the neuropathology^[80]^.

It has been reported that neuron-derived exosomes have the ability to confer conformational changes to extracellular Aβ, converting these molecules into non-toxic fibrils which promote uptake by microglia^[81]^. Secretion of these neuronal exosomes appears to be regulated by neutral sphingomyelinase 2 and sphingomyelin synthase 2 (SMS2). SMS2 siRNA enhanced exosome secretion and Aβ uptake into microglia and decreased extracellular Aβ^[81]^. Studies *in vivo* have shown that neuroblastoma-derived exosomes injected into mouse brain trapped Aβ and facilitated the internalization of Aβ into microglia. Continuous injection of these exosomes into amyloid-β precursor protein transgenic mice significantly reduced Aβ and Aβ-mediated synaptotoxicity in the hippocampus. Further studies revealed that glycosphingolipids that are highly distributed on these exosome membranes are critical for the Aβ binding^[82]^. Another line of study showed that N2a cell-derived exosomes could rescue Aβ-mediated disruption of synaptic plasticity via trapping Aβ with cellular prion protein^[83,84]^. Glycosphingolipids on these exosome are important for binding and sequestering Aβ^[85]^. All these studies suggest that exosomes may play an important role in the nervous system. Additionally, studies have suggested that exosomes contain a variety of components that produce neuroprotective effects such as neprilysin^[86]^ and insulin-degrading enzyme that are important for Aβ degradation^[87]^.

Other studies have provided controversial results which suggest that exosomes might play complicated roles in the development of AD. For example, in APPxPS1 transgenic AD mouse model, intracellular Aβ was found to be colocalized with raft marker flotillin-1, suggesting that Aβ accumulated within multivesicular bodies^[88]^. A minute fraction of Aβ was subsequently released into extracellular space in association with exosomes^[89]^. Similarly, other studies have shown that amyloid precursor protein (APP), APP-C-terminal fragments, and amyloid intracellular domain were all secreted from exosomes in differentiated neuroblastoma and primary neuronal culture cells^[90]^. In HEK-293-derived exosomes, Holo-APP, Presenilin and APP C-terminal fragments were all detected, and secretion of total APP C-terminal fragments was higher in exosomes derived from retromer deficient cells^[91]^. In addition, intraperitoneal injection of GW4869, a neutral sphingomyelinase 2 inhibitor, significantly decreased brain ceramide, exosome secretion from brain and serum exosome levels, as well as Aβ1-42 plaques in *5XFAD* mice^[92]^. In contrast, feeding female mice with ceramide showed a higher load of plaque burden and exosome secretion^[93]^,suggesting an association with exosome levels and Aβ accumulation in plaques. Furthermore, APP, BACE1, PSEN1, PSEN2 and Adam10, and many proteases that have the capacity to splice APP, have also been reported to be released from exosomes^[94]^. Thus, exosomes represent a novel pathway for APP processing and secretion, and amyloid deposition in AD brain. Interestingly, current research revealed that while during early stage of AD, activation of microglia produced protective effect by increasing phagocytosis and Aβ clearance^[95–97]^; during late stage of AD, microglia increased the release of exosomes or EV that contained soluble toxic Aβ and facilitated the progression of AD^[97,98]^.

Due to the unique characteristics of exosomes in that they recapitulate the features of the originating cells and are able to cross the blood brain barrier, their contents can serve as potential biomarkers for diagnosis and monitoring treatment and progression of AD. Tau^[99,100]^, and phosphorylated Tau have been detected in exosomes isolated from AD patients^[101]^, and can potentially serve as biomarkers for early diagnosis of AD, although further investigation is required to establish this connection. Furthermore, recent research has shown that serum-derived neuronal exosomes might be a potential biomarker for diagnosis and clinical monitoring of AD^[102,103]^. The use of exosomes as a delivery system for therapeutic drugs has also been extensively studied. Intranasal administration of exosome encapsulated drug led to rapid distribution of drugs into the brain^[104]^, indicating the possibility that exosomes can cross the blood brain barrier bi-directionally. Indeed, a large number of studies have shown that injection of exosomes as a drug delivery system could reduce Aβ and other relevant pathological changes^[97]^.

### Role of exosome in Parkinson’s disease

Parkinson’s disease (PD) is one of the most common neurodegenerative disorders affecting millions of people worldwide. The pathological hallmark of PD is the presence of Lewy bodies which contain misfolded α-synuclein (α-syn) that tends to aggregate, resulting in progressive loss of dopaminergic neurons in substantia nigra and striatum^[105]^.

Studies showed that Lewy bodies are initially found in the peripheral tissues, and then gradually spread to the brain stem, and eventually to cerebral cortex, suggesting PD progressed into the central nervous system from peripheral tissues, similar to prion-like disease^[106]^. Interestingly, studies revealed that exosomes play a critical role in the propagating and progression of PD^[107]^. First, exosomes have been found to be a carrier that can deliver pathological proteins: both newly synthesized and aggregated forms of α-syn could be released through unconventional, endoplasmic reticulum/Golgi-independent exocytosis. Intravesicular α-syn has a greater tendency to aggregate than the cytosolic protein. This secretion was enhanced by proteasomal and mitochondrial dysfunction associated with PD^[108]^. Further studies revealed that synaptic vesicles that contain α-syn could be sorted into early endosomes through Golgi or clathrin-mediated endocytosis^[109]^, and then transported into MVBs and fused with membrane to secrete the exosomes^[110]^. Alternatively, α-syn could also be sorted into the recycling endosome system and exocytosed as secretory granules^[111]^. Exosomes derived from α-synuclein producing cells, are released in a calcium-dependent manner. Studies have also shown that exosomes contribute to the formation of aggregation of α-syn: monitoring the aggregation kinetics with thioflavin T fluorescence revealed that exosomes facilitated the process by providing a catalytic environment for nucleation^[112]^. Quantification of cerebrospinal fluid (CSF) exosome numbers and α-syn content from PD patients revealed a correlation with the severity of cognitive impairment. Interestingly, incubation of exosomes derived from CSF of patients with PD and Lewy body dementia induced oligomerization of soluble α-synuclein in recipient cells in a dose-dependent manner. One hypothesis is that a pathogenic species of α-syn in these exosomes could induce oligomerization of soluble α-syn in the recipient cells to confer disease pathology^[113]^. It has also been suggested that exosome-mediated release of toxic forms of oligomeric α-syn, which is more easily taken up by recipient cells than free α-syn may be a mechanism for clearing toxic α-syn oligomers when autophagy is insufficient^[114]^.

Moreover, recent studies revealed that exosomes originated from the central nervous system could cross the blood brain barrier and carry the pathologic proteins into the blood^[115]^. Therefore, the cargo of serum/plasma-derived exosomes from patients with PD has been under extensive study as containing promising biomarkers in PD pathogenesis and clinical progression^[116–119]^.

It is noteworthy that exosomes are currently exploited as a drug delivery vehicle for treating PD. Several studies have demonstrated significant neuroprotective effects using exosome-based delivery system in *in vitro* and *in vivo* models of PD^[120–122]^. For example, intranasal administration of catalase-loaded exosomes effectively protected dopamine neurons in the substantia nigra pars compacta against oxidative stress in PD mouse brain^[122]^; and intravenous treatment with dopamine-loaded serum-derived exosomes also produced significant effects in PD mouse models^[122]^. However, the use of exosome delivery of therapeutics to treat PD remains challenging.

### Role of exosomes in Prion disease

Prion disease is a fatal neurodegenerative disease in humans and animals, caused by infectious abnormal microscopic protein particles known as prions. Prion disease is primarily characterized by assemblies of misfolded beta-sheet prion proteins in the brain and a rapid decline in cognition and cerebral and cerebellar functions^[123]^. Although the mechanism of prion transmission still remains unclear, studies have shown that misfolded prion proteins are associated with exosomes, and these exosomes could spread the disease^[124]^. Furthermore, studies showed that infection of N2a neuroblastoma cells with prions associated with scrapie could induce the release of prion proteins into the medium, predominantly via exosomes^[125]^. Knockdown of HRS/Vps27, a subunit of ESCRT-0 or TSG101-ESCRT-I subunit in Mov 127S cells significantly reduced accumulation or release of infective prion, suggesting that ESCRT-dependent and independent transmission mechanisms are both involved in the regulation of exosome-mediated release of prion proteins^[126]^. Stimulation of exosome release with monensin increased prion infectivity; by contrast, inhibition of exosome release with GW4869 decreased prion spreading^[127]^. Studies have also revealed that exosomes derived from neurons infected by prion could infect normal neurons and initiate prion propagation. In addition, these exosomes could induce prion disease when inoculated into mice. Interestingly, these prion proteins were found to have undergone N-terminal modification and selection of specific glycoforms for incorporation into exosomes^[128]^. In line with these findings, it was also reported that exosomes derived from infected mice could spread prion disease^[129]^. Recent studies also revealed that some specific miRNAs such as miR-146a, miR-29b found within exosomes from prion-infected cells may play important roles at various stages of spreading of prion disease^[130]^. All the data support that exosome potentially contributes to the rapid colonization in the development of prion disease.

### Role of exosomes in amyotrophic lateral sclerosis

Amyotrophic lateral sclerosis (ALS) is a fatal neurodegenerative disease in humans, which is characterized by progressive muscle atrophy due to the loss of motor neurons. Approximately 10% of ALS patients are familial, and the rest of 90% are sporadic. Both environment and genetic factors such as mutation of superoxide dismutase-1 (SOD1) and nuclear TAR DNA-binding protein 43 (TDP-43) have been shown to be involved in the pathogenesis of ALS^[131]^. A common pathologic feature of ALS is the aggregation of misfolded cytoplasmic proteins, for instance, TDP-43, ubiquilin 2 and SOD1^[132]^. TDP-43 is an RNA/DNA binding protein that regulates RNA transcription and DNA repair. Hyperphosphorylated and ubiquitinated TDP-43 has been reported to contribute to the development of ALS^[133]^. Strikingly, recent studies have revealed that aggregated TDP-43 or SOD1 proteins could be transported by exosomes to recipient cells to induce neurotoxicity^[134,135]^. In clinical studies, TDP-43 levels have been reported to be much higher in exosomes derived from frozen post-mortem temporal cortices of patients with sporadic ALS, compared with controls^[136]^. A clinical 3- and 6-month follow up study also showed exosomal TDP-43 levels were much higher in ALS patients compared with the control group^[137]^. *In vitro*, TDP-43 is secreted via exosomes in Neuro2a cells, and inhibition of exosome secretion exacerbates the aggregation of TDP-43. In addition, inhibition of exosome secretion also worsens the phenotype of TDP-43^A315T^ transgenic mice^[136]^. Other studies showed that exosome-induced cytokine secretion is compromised in CD14++ monocytes from ALS patients, and this abnormality is modulated by exosomal TDP-43, suggesting that exosomal TDP-43 contributes to the impaired neuroinflammatory reaction in ALS pathogenesis^[134]^.

With distinct advantages, exosomes can also be used as therapeutic delivery carriers. Exosomes isolated from adipose-derived stem cells have been shown to restore mitochondrial complex I activity, efficiency of electron transfer system and membrane potential in an *in vitro* model of ALS, NSC-34 cell line overexpressing human mutated SOD1, suggesting a potential therapy for ALS using such exosomes^[138]^.

### Role of exosomes in Huntington’s disease

Huntington’s disease (HD) is a progressive autosomal dominant neurodegenerative disease that is characterized by cognitive impairment and involuntary choreiform movements. Pathologically, it is caused by CAG expansion in exon 1 in Huntingtin gene that leads to production of mutant huntingtin (mHtt). Emerging research has revealed that the mutated products, polyglutamine protein could lead to severe neuronal toxicity, and CAG repeat length is positively associated with clinical symptoms^[139]^. To date, studies have implied that exosomes are involved in the pathogenesis and propagation of Huntington disease^[140]^. When SH-SY5Y cells were cultured with conditioned medium from HEK cells that overexpress GFP, GFP-mHtt-Q19 or GFP-mHtt-Q103, the exogenous mHtt proteins were detectable within SH-SY5Y cells after 5 days of exposure. In addition, after co-culturing mouse neural stem cells with exosomes derived from fibroblast from HD patient carrying the 143 CAG repeat (HD143F) for 4 days, mHtt aggregates were detected within the neurons, suggesting mHtt could propagate from cell to cell through internalizing exosomes that contain pathological proteins. Furthermore, intraventricular injection of exosomes isolated from HD143F, resulted in the Huntington-like phenotype in mice, and mHtt was detected in the striatum^[141]^. In another study, both *in vivo* and *in vitro* data suggest that extracellular vesicles can transfer toxic trinucleotide repeat RNAs between cells and trigger the manifestation of HD-related behaviors and pathology in mice; however, activity of exosomes or cell-type specificity was not fully evaluated^[142]^. These findings support the hypothesis that exosomes contribute to the HD progression by transferring toxic proteins or RNAs from one cell to another. Data have revealed that certain types of circulating microRNAs were up or down regulated in patients with HD, but exosome-derived microRNAs as biomarkers are still under investigation^[143,144]^. Moreover, recent studies showed that injection of exosome-delivered miR-124 into R6/2 transgenic HD mice reduced the RE-1 Silencing Transcription Factor, which is involved in the development of HD^[145]^. In addition, infusion of hydrophobically modified Htt-hsiRNA-loaded exosomes into mouse striatum resulted in significant bilateral silencing of about 35% of Huntingtin mRNA^[146]^. Thus, the potential use of exosomes as a route for delivering various siRNAs to the brain to suppress expression of mHtt or other relevant regulatory proteins offers another approach to treating HD.

### Role of exosomes in epilepsy

Epilepsy is a neurological disorder that is characterized by abnormal electrical discharge of brain neurons^[147]^. Status epilepticus can lead to neuron damage and gliosis^[148]^. Emerging studies have suggested that microvesicles such as exosomes could be released following brain injury or stimulation and serve as a biomarker for epilepsy. For example, status epilepticus induced by intra-amygdala kainic acid led to upregulation of both ESCRT-dependent and -independent signaling pathways and thus increased exosome release in mice. This effect lasted for a long time and the enhanced secretion of exosomes was still detectable 2 weeks after status epilepticus^[149]^. In addition, studies from both animals and human have implied that certain types of exosomal miRNA are highly associated with epilepsy. In a rat model of chronic temporal lobe epilepsy, miR-346 and miR-331-3p were found to be decreased in extracellular vesicles of the forebrain^[150]^. Besides, a clinical study involving 40 patients with mesial temporal lobe epilepsy with hippocampal sclerosis (mTLE-HS) showed that two exosomal miRNAs were upregulated, while 48 miRNAs were downregulated. Among these candidates, exosomal miRNA-8071 was reported to have the sensitivity of 83.33% and the specificity of 96.67% for diagnosis of mTLE-HS^[151]^. In another study, exosomal circulating miRNAs, such as miR194-2-5p, miR15a-5p, miR-132-3p, and miR-145-5p, have been reported to be potential biomarkers in patients with focal cortical dysplasia and refractory epilepsy^[152]^. Interestingly, intranasal administration of A1-exosomes derived from human bone marrow-derived MSCs rescued neuron loss, inflammation and neurogenesis, as well as alleviated compromised memory and cognitive capacity in mice which typically occur after status epilepticus^[153]^. These studies demonstrate that epilepsy could alter exosome release and its miRNA content, which could be a potential biomarker for clinical diagnosis. Further studies in exosomes will be needed to identify the distinct types of epilepsy subtype to determine the specific miRNA pathophysiological significance for epileptogenesis.

### Role of exosomes in multiple sclerosis

Multiple sclerosis (MS) is a chronic inflammatory demyelinating disease of the central nervous system^[154]^. Currently, the etiology of MS remains elusive, and the diagnosis mainly relies on clinical symptoms. Thus, earlier diagnosis and effective clinical intervention are very important for improving patient outcomes. Recent studies have found that exosomal contents such as myelin oligodendrocyte glycoprotein^[155]^, sphingomyelinase (SMase)^[156]^, and a variety of microRNA^[157–160]^ are potential diagnostic biomarkers for MS. In addition, Schwann cell-derived exosomes contain a variety of neuroprotective proteins and anti-inflammatory factors^[161]^ that play critical roles in MS via regulating myelin membrane biogenesis and providing trophic factors required for myelin maintenance^[162]^. For instance, exosomes which contain myelin and protective proteins against stress, were released from oligodendrocytes into the extracellular space in a calcium dependent manner^[163]^; Schwann cell-derived exosomes can improve axonal regeneration after axotomy^[164]^, and increase nerve activity^[165]^. Exosomes from adipose-derived mesenchymal stem cells, bone marrow-derived mesenchymal stem cells, and umbilical cord stem cells have shown potential therapeutic effects in protecting oligodendrocyte and promoting neurite outgrowth and nerve regeneration^[166–168]^.

### Function of exosomes in stroke

Stroke has been increasing during past few decades and has become one of the major life-threatening medical conditions around the world. Thus, early diagnosis and effective monitoring of recovery phases are critical for the management of stroke patients. Compared with most biomarkers obtained from blood and body fluids, exosomes have an advantage due to their high heterogeneity^[7]^ which reflects the pathophysiological conditions of the cells from which they originate, and thus their cargo are potential biomarkers for diagnosis and clinical evaluation. Studies have shown that exosomes can cross the blood brain barrier and enter peripheral blood and cerebral spinal fluid after stroke^[169]^. In addition, exosomes orchestrate a complicated process after stroke involving nerve regeneration, angiogenesis, neurogenesis, remodeling of immune response, neuronal plasticity and axon dendrite outgrowth^[170]^. Studies have shown that endothelial cell-derived exosomes can promote the differentiation of neural progenitor cells into oligodendrocytes for myelination; neuron and neuronal progenitor cell-derived exosomes can regulate peripheral immune response; pericyte-derived exosomes can facilitate neurogenesis; circulating endothelial progenitor cell-derived exosomes can facilitate angiogenesis by interacting with cerebral endothelial cells^[7]^. Further studies revealed that mesenchymal stromal cell-derived exosome enhanced neurite branch and length in rat cultured neurons after middle artery occlusion^[171]^. Exosomal miR-126 and miR-124 were also reported to be involved in the angiogenesis^[9,172]^ in rats and neurogenesis in mice after stroke^[173]^. However, exosomes also produce some adverse effects in peripheral organs after stroke, such as increasing pro-inflammatory cytokines and activating T and B lymphocytes, thus effecting heart^[174]^, kidney^[175]^, and digestive intestine system^[7]^.

Extensive studies have shown that stroke could induce a variety of changes in the contents of exosomes released from central nervous system. For example, next generation sequencing analysis showed that human neural stem cell-derived miroRNAs were altered by hypoxic condition^[176]^. Data from both human and animal models suggested that certain types of exosomal cargoes were altered: In animal models, plasma-derived exosomal rno-miR-122-5p was significantly downregulated and rno-miR-300-3p upregulated in ischemic rats^[177]^. In clinical studies, proteome analysis of microvesicles from plasma of patients with lacunar infarction revealed that brain-related proteins such as myelin basic protein, focal adhesion and coagulation related proteins were upregulated, and albumin was downregulated in patients with adverse outcomes compared with matched controls^[178]^. Analysis of plasma EV from patients with manifest vascular disease showed elevated protein cystatin C and CD14 levels correlated with white matter lesions and progression of brain atrophy^[179]^. In patients with acute ischemic stroke, the serum exosome levels of miR-9 and miR-124 were both elevated compared with healthy controls, and positively correlated with National institute of Health Stroke Scale scores (NIHSS), infarct volumes and IL-6 levels^[180]^. Plasma-derived exosome miR-422a and miR-125b-2-3p were both decreased during the subacute phase of ischemic stroke, with miR-422a increased in the acute phase in comparison with controls^[181]^. In addition, exosomal miRNA such as miR-223, miR-21-5p and miRNA-30a-5p were also reported to be highly related with occurrence and severity of stroke in several clinical studies^[182,183]^. These results suggest that designing a multiplex platform to assay for multiple biomarker molecules in exosomes known to be associated with stroke might be a promising approach for diagnosis and clinical progress evaluation of stroke patients, especially with the advancement of exosome isolation and purification techniques.

### Role of exosomes in traumatic brain injury

Traumatic brain injury (TBI) often leads to injury-induced death and disability around the world^[184]^. After TBI, brain parenchymal damage and hemorrhage and compromised blood-brain barrier, as well as associated inflammation, oxidative stress and cell death contribute to the TBI-induced pathological alterations and dysfunction. As a critical player in cell communication, exosomes have been proposed to be able to carry specific biomarkers during traumatic brain injury and can serve in early diagnosis of concussion and monitoring of clinical progress^[185]^. Recent studies implied that certain components such as miR-124-3p in microglial exosomes were upregulated significantly after TBI and exerted anti-inflammatory function and promoted neurite outgrowth^[186]^. In another study in veterans with mild traumatic brain injury, elevated exosome-derived neurofilament light chain was observed, even years after injury^[187]^. Studies of serum-derived neuronal exosomes from patients with acute TBI and chronic TBI showed that proteins associated with neuronal functions were significantly increased in acute TBI, while neuropathological proteins were up-regulated in both acute and chronic TBI. These results suggest that cargo in serum-derived neuronal exosomes could act as potential biomarkers for clinical diagnosis^[188]^. Additionally, the capacity of exosomes to cross the blood-brain barrier offer a potentially effective therapeutic approach in treatment of patients with TBI^[189]^.

### Roles of exosomes in neuropsychiatric disorders

Neuropsychiatric disorders such as major depression and schizophrenia are associated with certain changes of brain structures and neurotransmitters. Although the molecular mechanism is not fully understood, emerging studies suggest that miRNAs enriched in exosomes may be key factors in the development of neuropsychiatric disorders^[190–194]^. Acting as a complicated mediator of cell communications, alterations of exosomal components have been identified in patients with neuropsychiatric disorders^[10]^. One analysis of exosomal miRNAs from frozen postmortem prefrontal cortices of patients revealed that miR-497 was significantly elevated in schizophrenia, and miR-29c increased in bipolar disorders in comparison with control^[195]^. Genome-wide analysis of miRNAs from serum exosomes, with subsequent bioinformatic predictions and validations, has also indicated miRNA dysregulation in schizophrenic patients^[196]^. Of all the miRNAs, hsa-miR-206 was the most upregulated in these patients. Hsa-miR-206 has been reported to interact with *BDNF* mRNA directly, leading to the decreased expression of this gene and compromised cognitive function in mice^[197]^. In another study, in patients with depression, 12 miRNAs that regulate the neurotrophin pathway were found to be increased, and 20 miRNAs that control apoptosis, cell growth, immune and hypoxia activity were downregulated^[198]^. A recent study has revealed that exosomal miR-139-5p is significantly increased in patients with major depressive disorder in comparison with controls, suggesting it might be a potential biomarker for this disorder^[199]^.

To date, studies in the role of exosomes in neuropsychiatric disorders are very limited. These findings have opened up challenging possibilities of uncovering the function of exosomes and molecules associated with them in mental disorders.

### Exosomes in brain tumors

**Figure 3.**
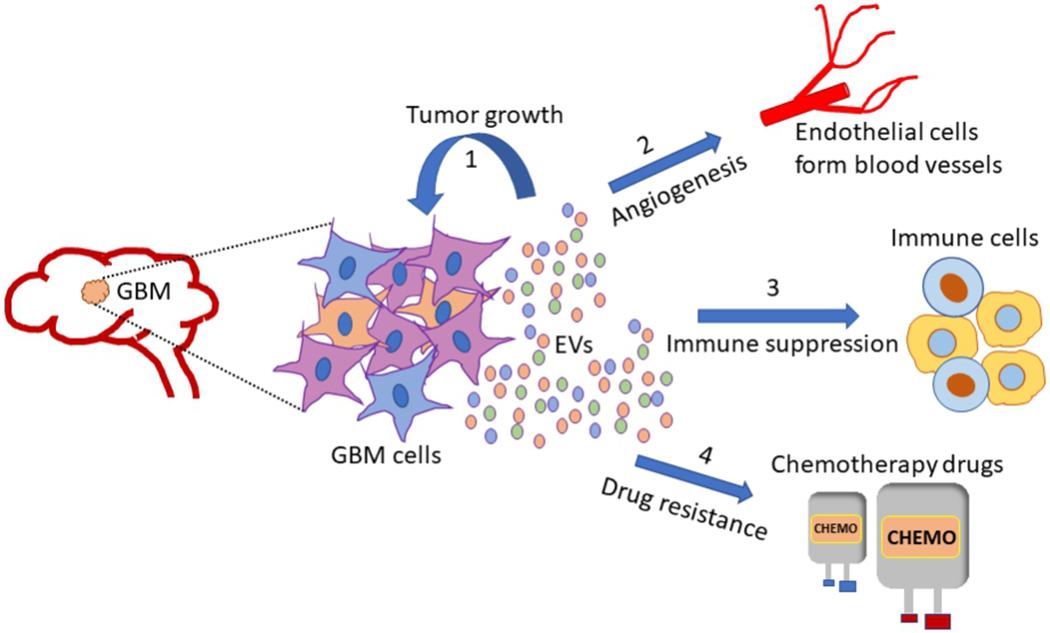
Glioblastoma multiforme (GBM) is one of the most aggressive tumors of the adult brain. Cells that make up the GBM tumor, release extracellular vesicles (EVs), which mediate the transfer of vital cues between tumor cells and the surrounding microenvironment. GBM tumor mass is highly heterogenous comprising differentiated tumor cells and glioma stem cells (GSCs). GSC derived EVs are particularly important players in sustaining glioma growth and invasion. Depending on their cell of origin, GBM EVs deliver unique cargo such as proteins, nucleic acids and lipids to recipient cells, to possibly alter their gene expression profile and phenotypes, and in the process favor malignant transformation. Some critical functions attributed to GBM EVs include (1) supporting tumor growth and survival; (2) promoting angiogenesis by regulating gene expression in endothelial cells; (3) mediating immune evasive phenotype changes in tumor associated immune cells: T cells, macrophages and microglia; (4) inducing resistance to chemotherapy drugs/radiation therapy. GBM: Glioblastoma multiforme; EVs: extracellular vesicles.

Glioblastoma multiforme (GBM) is the most aggressive and common primary tumor of the adult brain, with median survival of less than 15 months from diagnosis^[200]^. Regardless of patients receiving rigorous standard of care, such as surgical resection alongside chemotherapy and/or radiotherapy, this rare astrocytoma has very poor prognosis^[201,202]^. Among the heterogenous cell populations that form the GBM tumor mass are the cancer stem cells, which contribute to therapy resistance, tumor growth and recurrence^[203–206]^. Recent reports have^[207]^ suggested that EVs including exosomes mediate critical bilateral communication between the tumor cells and their microenvironment to sustain the growth of malignant GBM. A longitudinal time-lapse imaging study showed that glioma cells have crosstalk with non-glioma cells such as glial cells, neurons and vascular cells through EVs, to alter the tumor microenvironment and promote glioma growth *in vivo*^[208]^. GBM derived EVs are known to facilitate angiogenesis, tumor progression and invasion, drug resistance and immune regulation^[209–212]^ [Figure 3]. Moreover, various GBM exosomal cargoes are involved in mediating these processes [Table 2].

Hypoxia within the GBM microenvironment promotes neo-angiogenesis, as a means to supply oxygen and nutrients to the rapidly growing tumor cells. VEGF-A carrying EVs secreted by glioma stem cells (GSC), stimulates endothelial cells to proliferate, migrate and form tubular structures, promoting vasculature^[213]^. In addition, GSC exosomes can transfer miRNAs such as miR-21 and miR-26a to endothelial cells, to upregulate VEGF expression and support GBM angiogenesis^[214,215]^. Studies using clinical samples have shown that microvesicles derived from CSF of GBM patients upregulate proliferation of cultured endothelial cells through AKT/beta-catenin pathway^[216]^. Oncogenic EGFRvIII and tissue transglutaminase are reportedly other protein factors transferred through EVs, which are known to induce mitogenic and/or angiogenic signaling in recipient cells^[217,218]^. Interestingly, exosome-mediated delivery of long non-coding RNAs such as lncCCAT-2 and lncPOU3F3 can also enhance vascularization of GBM^[219,220]^. On the contrary, miR-1 enriched glioma EVs have been implicated in suppressing angiogenesis and tumor growth^[221]^. Increased growth and aggressiveness of advanced stages of GBM is associated with the phenotypic transition from proneural to mesenchymal subtype. GSC EVs contribute towards this process, by way of transferring mRNAs, miRNAs and other regulatory RNAs, and transcription factors, which can possibly reprogram the recipient cells, alter their epigenetic signatures and render the GBM microenvironment more permissive to malignant transformation^[211,222,223]^. EV-mediated crosstalk in GBM involves the interaction between a chemokine receptor CCRs on recipient cells and the glycans on the EV surface, with the CCL1 ligand acting as a bridging molecule^[224]^. RNA-seq and DNA methylation analyses showed that pro-angiogenic miRNA such as miR-9-5p transferred via GSC EVs can reprogram human brain endothelial cells *in vitro* to induce angiogenesis, by distinct pathways compared to those activated by vascular growth factors^[225]^. Similar molecular profiling studies conducted earlier using GSC EVs had revealed that the molecular subtypes and functional state of GSCs determine the tumor regulatory effect of EVs^[226,227]^.

GBM cells interact with the surrounding astrocytes to modulate tumor growth and survival. In a study using patient tumor derived cell lines, it was shown that GBM EVs can transform normal human astrocytes to a pro-tumorigenic phenotype, exhibiting increased production of growth factors, chemokines and cytokines, to support in vitro growth of GBM cells^[207]^. GBM EVs regulate tumor signaling pathways such as p53 and c-MYC in astrocytes to induce a senescence associated secretory phenotype, to favor tumor progression^[228]^. Moreover, EVs derived from GBM cells were shown to induce podosome formation, ECM degradation and increased migration of astrocytes^[207,228]^. Astrocytes cultured with GBM EVs show enhanced secretion of immunosuppressive cytokines such as CSF2 and interleukins 4, 10 and 13, thus providing a tumor supportive microenvironment. EVs secreted by irradiated GBM cells have enhanced presence of CD147, which in turn stimulates increased MMP9 release from recipient astrocytes, suggesting the contribution of astrocyte signaling in promoting GBM invasiveness, particularly in response to ionizing radiation^[229]^. A recent study reported that GSC EVs induce metabolic reprogramming of pre-transformed astrocytes to enhance proliferation, self-renewal and tumor growth in a mouse allograft model^[230]^.

Immune suppression fosters aggressive transformation of brain tumor. The molecular cargo transferred by GBM EVs can influence the status of tumor-associated macrophages or myeloid cells (TAMs)^[231]^. GBM EVs induce the *in vitro* differentiation of peripheral blood derived monocytes to anti-inflammatory M2-macrophages, which exhibit enhanced phagocytosis and secretion of IL-6 and VEGF, to support immune evasion of glioma^[232]^. Peripheral blood analyses of GBM patients signified the role of tumor-derived exosomes in promoting an immune evasive Th2 bias, and their ability to induce CD163 (a macrophage marker) expression on normal astrocytes^[233]^. EVs carrying miR-21 released by GBM cells were shown to target the expression of Btg2, an anti-proliferative protein in recipient microglia, subsequently reprogramming these cells to support tumor progression^[231,234]^. STAT3 pathway proteins present within GSC EVs including exosomes, also mediate immune suppressive changes of monocytes including their phenotype change to M2-macrophages, cytoskeletal reorganization, and upregulation of PD-L1 ligand, which binds to PD1 to inhibit T-Cell activation^[235–237]^.

Studies addressing the mechanism of resistance to Temozolomide (TMZ), an alkylating agent used as the standard of care for glioma, have uncovered the possible influence of EVs in the process. Using a microfluidic chip-based analysis, it was found that sera-derived EVs from GBM patients (small cohort study) are enriched in mRNA levels of MGMT (O6-methylguanine DNA methyltransferase) and APNG (alkylpurine-DNA-N-glycosylase), primary DNA repair enzymes involved in inducing chemoresistance^[210]^. miR-9 is upregulated in exosomes released from TMZ resistant glioma cell lines and is implicated in increasing MDR1 (multidrug resistance mutation 1) expression and repressing Patched (PTCH1), Sonic Hedgehog receptor to confer chemoresistance^[238]^. Transfer of anti-miR-9 through MSC exosomes to GBM cells was shown to impart chemosensitivity and reverse multidrug transporter expression^[239]^. Analysis of clinical samples has revealed that Pacritinib, a STAT3 inhibitor can potentially overcome TMZ resistance by reducing miR-21 enriched exosomes secreted by GBM-associated macrophages^[240]^.

EV cargo can be potential biomarkers for GBM diagnosis and progression. EGFRvIII is found in high levels within GBM EVs, and hence can be used as a potential biomarker^[211]^. A study conducted using clinical samples from GBM patients undergoing tumor resection, reported that CSF derived EVs can be developed as a diagnostic tool to assess EGFRvIII positive GBM status accurately^[241]^. CSF EVs of GBM patients have also been found to be enriched in miR-21 compared to non-oncologic patients, suggesting the potential use of CSF derived EV miR-21 as another biomarker for GBM prognosis^[242,243]^. In addition, RNA/proteins with growth promoting functions such as TrkB (neurotrophin tyrosine kinase receptor-1), Timp1 (NF-κB target gene) and CLIC1 (circulatory protein), and PTEN, a tumor suppressor protein, enriched within EVs are some other potential prognostic biomarkers for GBM^[209]^.

## CONCLUSION AND PERSPECTIVES

Emerging research in the last decade suggests that exosomes and EVs are critical players in regulating physiological and pathological processes in the brain. Exosomes and EVs mediate intercellular communication by trafficking of biomolecules such as proteins, lipid, mRNA, and miRNA.

Circulating exosomes have vast potential in being developed as a source of biomarkers for various neurodegenerative disorders and brain tumors, and as vehicles for drug delivery. Neurotoxic proteins associated with AD, PD and prion disease, such as Aβ, Tau, α-synuclein and PrP respectively are encapsulated and transferred through exosomes. Oncogenic proteins (EGFRVIII, TrkB, Timp1) and miRNAs (miR-21, miR-9) carried as exosomal cargo can reprogram recipient cells in the tumor microenvironment to favor glioma progression. Besides targeting these exosomal proteins for therapeutics, the possibility of isolating exosomes readily from the circulating biofluids represents a novel and effective tool for non-invasive diagnosis and monitoring the status of various neurological conditions and glioma progression.

Knowledge of the fundamental aspects of exosome biology (exosome biogenesis, origin, cargo sorting and targeting to specific recipient cells) and the downstream signal transduction, is key to the application of exosomes for treatment of brain disorders. Although data have indicated that various exosomal proteins or miRNAs are altered during the development of neurodegenerative or other CNS diseases, precise signaling cascade or involvement is not well understood. This might be due to the limitation of isolation and characterization techniques of exosomes that fail to specifically capture and identify exosomes from specific cell type of origin, such as neurons or microglia from limited sample volume. Therefore, more specific exosome associated biomarkers and better isolation and purification techniques for capturing specific sub-populations of exosomes will greatly advance the ability to identify biomarkers.

Research advances in areas of exosome isolation, characterization, tissue targeting and understanding of their specific biological functions would allow exosomes to impact clinical therapy of neurological diseases. Indeed, the future prospect of developing the use of exosomes for delivery of functional cargo such as miRNA, siRNA and mRNA/proteins into the brain and other regions of the nervous system, such as in axonal regeneration, opens up exciting new avenues for drug delivery applications.

## Supplementary Material

video

## Figures and Tables

**Table 1.** Exosome cargo as biomarkers in neurodegenerative disorders

Name of disease	Exosome cargo	Pathology	Application	Ref.
Alzheimer’s disease	Aβ	Neuronal impairment	Early diagnosis	Saman *et al.*^[99]^,2012
Parkinson’s disease	α-syn	Neuronal damage	Early diagnosis Monitoring severity of cognitive impairment	Shi *et al.*^[116]^, 2014 Stuendl *et al.*^[113]^,2016 Si *et al.*^[117]^,2019 Jiang *et al.*^[118]^,2020 Niu *et al.*^[119]^,2020
Amyotrophic lateral sclerosis	TDP-43	Neuronal inflammation and damage	Early diagnosis	Chen *et al.*^[137]^,2020

**Table 2.** Function of EVs/exosomes in regulating glioma

Types of exosomal cargo	Parental cell	Recipient cell	Function	Ref.
VEGF-A	GSC	Endothelial cell	Promote angiogenesis	Treps *et al.*^[213]^,2017
miR-21, miR-26a, miR-9-5p	GSC	Endothelial cell	Promote angiogenesis	Sun *et al.*^[214]^,2017 Wang *et al.*^[215]^, 2019 Lucero *et al.*^[225]^, 2020
lncCCAT-2, lncPOU3F3	GBM cell	Endothelial cell	Promote angiogenesis	Lang *et al.*^[219]^,2017 Lang *et al.*^[220]^,2017
EGFRvIII, tissue transglutaminase	GBM cell	GBM cell	Support tumor growth	Al-Nedawi *et al.*^[217]^,2008 Antonyak *et al.*^[218]^,2011
CD147	Irradiated GBM cells	Astrocytes	Support tumor invasion	Colangelo *et al.*^[229]^,2020
STAT3 proteins	GSC	Monocytes	Immunosuppression	Gabrusiewicz *et al.*^[235]^,2018 Grimaldi *et al.*^[236]^,2019 Ricklefs *et al.*^[237]^,2018
miR-21	GBM cells	Microglia	Immunosuppression, tumor growth	Abels *et al.*^[234]^, 2019 Van der Vos *et al.*^[231]^, 2016
*MGMT, APNG*	GBM cells	GBM cells	Chemoresistance	Shao *et al.*^[210]^,2015
miR-9	GBM cells	GBM cells	Chemoresistance	Munoz *et al.*^[239]^, 2013 Munoz *et al.*^[238]^,2015
miR-21	Tumor associated macrophages	GBM cells	Chemoresistance	Chuang *et al.*^[240]^,2019
